# Comparative Transcriptome Analysis of Recessive Male Sterility (RGMS) in Sterile and Fertile *Brassica napus* Lines

**DOI:** 10.1371/journal.pone.0144118

**Published:** 2015-12-10

**Authors:** Cunmin Qu, Fuyou Fu, Miao Liu, Huiyan Zhao, Chuan Liu, Jiana Li, Zhanglin Tang, Xinfu Xu, Xiao Qiu, Rui Wang, Kun Lu

**Affiliations:** 1 Chongqing Engineering Research Center for Rapeseed, College of Agronomy and Biotechnology, Southwest University, Tiansheng Road 2, Beibei, Chongqing 400716, China; 2 Engineering Research Center of South Upland Agriculture of Ministry of Education, Southwest University, Beibei, Chongqing 400716, China; 3 Food and Bioproduct science, University of Saskatchewan, 51 Campus Drive, S7N 5A8, Saskatoon, SK, Canada; 4 Agriculture and Agri-Food Canada, Saskatoon Research Centre, 107 Science Place, S7N 02X, Saskatoon SK, Canada; Wuhan University, CHINA

## Abstract

The recessive genetic male sterility (RGMS) system plays a key role in the production of hybrid varieties in self-pollinating *B*. *napus* plants, and prevents negative cytoplasmic effects. However, the complete molecular mechanism of the male sterility during male-gametogenesis in RGMS remains to be determined. To identify transcriptomic changes that occur during the transition to male sterility in RGMS, we examined the male sterile line WSLA and male fertile line WSLB, which are near-isogenic lines (NILs) differing only in the fertility trait. We evaluated the phenotypic features and sterility stage using anatomical analysis. Comparative RNA sequencing analysis revealed that 3,199 genes were differentially expressed between WSLA and WSLB. Many of these genes are mainly involved in biological processes related to flowering, including pollen tube development and growth, pollen wall assembly and modification, and pollen exine formation and pollination. The transcript profiles of 93 genes associated with pollen wall and anther development were determined by quantitative RT-PCR in different flower parts, and classified into the following three major clades: 1) up-regulated in WSLA plants; 2) down-regulated in WSLA plants; and 3) down-regulated in buds, but have a higher expression in stigmas of WSLA than in WSLB. A subset of genes associated with sporopollenin accumulation were all up-regulated in WSLA. An excess of sporopollenin results in defective pollen wall formation, which leads to male sterility in WSLA. Some of the genes identified in this study are candidates for future research, as they could provide important insight into the molecular mechanisms underlying RGMS in WSLA.

## Introduction

*Brassica napus* (L.) (rapeseed) is an important worldwide source of vegetable oil and biofuel, and a nutrient-rich source of protein for livestock feed [1, 2]. The yield gain of rapeseed varieties is much lower than that of many classical crops, such as wheat, maize, and rice. Although rapeseed breeders around the world strive to develop robust hybrid rapeseed cultivars, they are unable to meet the demands of the increasing world population. Hence, high seed yield is an important breeding objective for this crop.

In flowering plants, pollen development and pollination affect the number of seeds formed and the quality and yield of crops. Male sterile plants are useful tools for hybrid seed production, as these plants are unable to self-pollinate and depend on pollen from another line for seed production. Several pollination control systems have been reported, including cytoplasmic male sterility (CMS) and genic male sterility (GMS)[3]. GMS, which offers advantages over CMS, such as complete male sterility and the absence of negative cytoplasmic effects, has been widely used in the production of hybrid rapeseed varieties [4]. Recessive genetic male sterility (RGMS), which is controlled by recessive gene(s), are easy to breed, since most lines can function as restorers. Therefore, RGMS has been widely used in *B*. *napus* programs in China. Examples of lines exhibiting RGMS include S45AB [5], 117AB [6], 9012AB [7, 8], 20118A [9], and 7365AB [10, 11]. Two near-isogenic lines (NILs), WSLA and WSLB, the latter of which exhibits RGMS, have been used for rapeseed heterosis and Yuyou 27 (No. 2012002), which is widely grown in China, is based on this system. Despite the commercial success of Yuyou 27, the mechanism underlying male sterility in this line was unclear.

**Fig 1:**
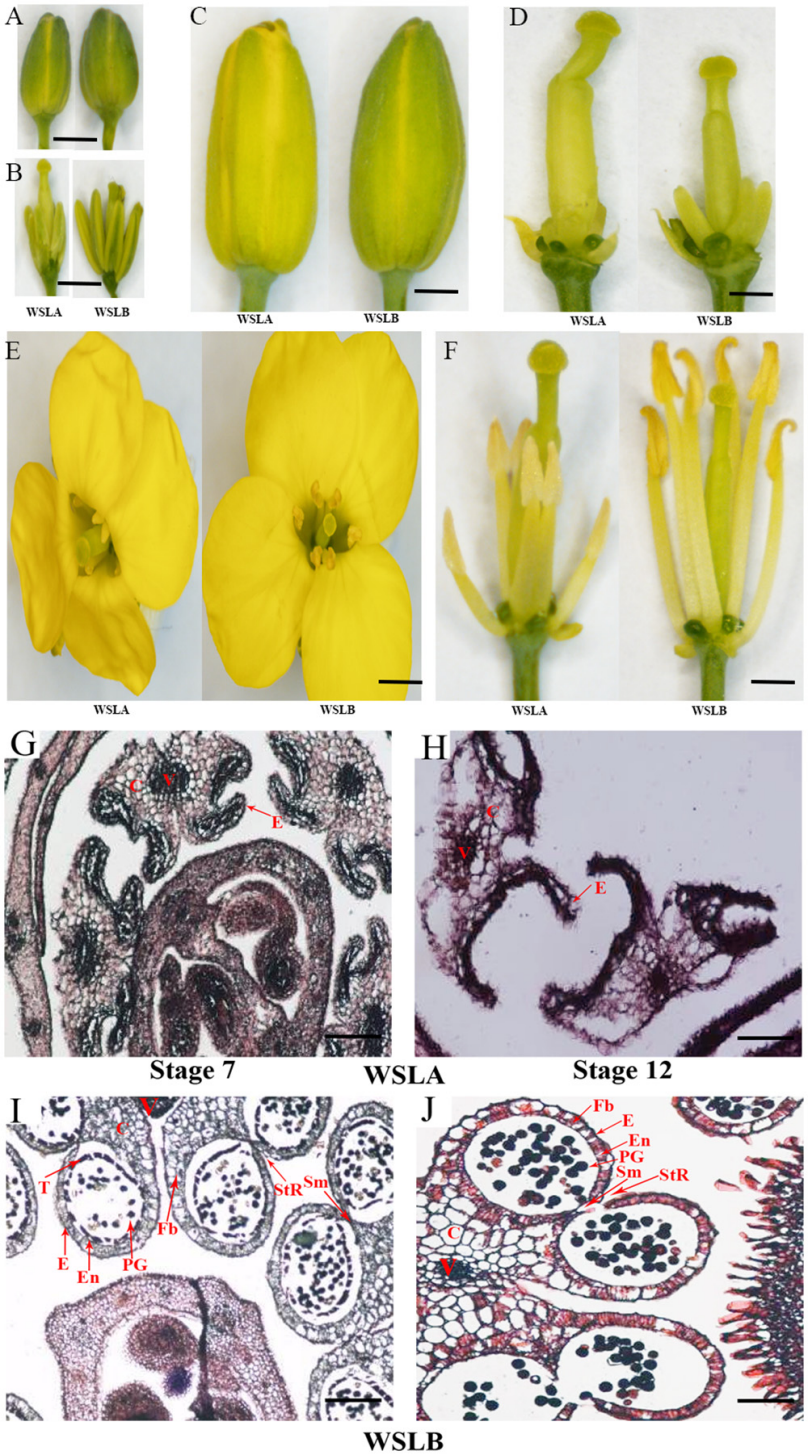
Phenotypic characterization of WSLA (Ste) and WSLB (Fer) floral buds. A-F, phenotype of sterile and fertile floral buds. G-J, Transverse sections through all of the buds. V, vascular region; C, connective region; E, epidermis; En, endothecium; Fb, fibrous bands; PG, pollen grains; Sm, septum; StR, stomium region; T, tapetum. A-F, Bar = 1.0 cm; G-J, Bar = 10 μm.

The mechanism underlying RGMS is extremely complex, as it involved many genes and metabolic pathways involved in pollen development and pollination. For example, the recessive genic male sterile line S45AB is controlled by two duplicate recessive genes, *Bnms1* and *Bnms2* [5]. The 9012A and 7-7365A lines have a similar inheritance model as S45AB, with sterility being controlled by two pairs of recessive duplicate genes (*Bnms3* and *Bnms4*) [12]. 7365AB, a RGMS system, is controlled by a recessive gene (*BnMs3*) and an epistatic gene (*BnRf*) in *B*. *napus*, and the expression of the recessive male sterility was related to the homozygosity *Bnrf* locus (*Bnrfrf*) in homozygous *Bnms3ms3* plants, resulting in male fertility [13]. Furthermore, *BnMs3* is essential for tapetal function and microspore development in *B*. *napus* [11, 13]. Unlike other sterile *B*. *napus* lines, however, the anthers of WSLA and WSLB showed prominent differences (Fig 1), indicating that male sterility may be controlled by an unknown gene in WSLA.

In *Arabidopsis thaliana*, numerous genes that are crucial for anther development have been elucidated. In the early stages of anther development, *SPOROCYTELESS* (*SPL*)/*NOZZLE* and *ROXY* are involved in archesporial cell differentiation [14–16]. *AtMYB103*, *Dysfunctional Tapetum 1* (*DYT1*), *Tapetal Development Function 1* (*TDF1*), *Aborted Microspore* (*AMS*), and *Male Sterility 1* (*MS1*) play important roles in tapetal development and programmed cell death [17–21]. Furthermore, transcriptome profiling revealed that *SPL*, *EMS1*, and *MS1* play crucial roles during the early stages of anther development in *Arabidopsis* [15, 22]. The exine is mainly composed of a complex biopolymer named sporopollenin, which consists of fatty acids and phenols [23]. *FACELESS POLLEN 1* (*FLP1*), *NEF1*, *MS2*, *Acyl-CoA Synthetase 5* (*ACOS5*), *CYP703A2*, *CYP704B1*, and *CYP704B2* were shown to be essential for the biosynthesis of sporopollenin during anther development [24–31]. Moreover, *LESS ADHESIVE POLLEN 3*, *LESS ADHESIVE POLLEN* 5, and *LESS ADHESIVE POLLEN* 6, that are involved anther-specific chalcone synthase (CHS) proteins, play roles in the biosynthesis of both fatty acids and phenols [32, 33]. In *Arabidopsis*, ABC transporters, such as WBC27, are essential for pollen wall development [34, 35]. *B*. *napus* and *A*. *thaliana* belong to the Brassicaceae family and share high levels of sequence similarity [36, 37]. However, a comprehensive understanding of anther development in *B*. *napus* male sterile and fertile lines is still lacking.

The WSLA is a recessive epistatic genic male sterile oilseed rape line that is characterized by short anthers and a lack of pollen grains on the anther surface of opened flowers. To elucidate the molecular mechanism governing the male sterility in this line, we generated two cDNA libraries from RNA extracted from WSLA and WSLB buds and sequenced these libraries using an Illumina Hiseq2000 platform. Comparative analysis of the expression profiles revealed that the expression of 3,199 genes (*P* < 0.01) differed significantly differences between WSLA and WSLB. Subsequently, 93 genes that were differentially expressed in the fertile and sterile lines were verified by reverse transcription quantitative PCR (RT-qPCR) using six components of flowers that are collected from WSLA and WSLB, respectively. These findings lay a solid foundation for further studies of the molecular mechanism underlying RGMS, and provide a framework for additional functional and pathway analyses.

## Materials and Methods

### Plant materials

Sterile (WSLA) and fertile (WSLB) near-isogenic lines (NILs) of *B*. *napus* were grown at the experimental station of the Rapeseed Engineering Research Center of Southwest University in Beibei, Chongqing, China. For transcriptome sequencing, total RNA from young flower buds (≤ 4 mm in diameter) of three independent plants was pooled, immediately frozen in liquid nitrogen, and stored at -80°C for RNA extraction.

### Tissue preparation and light microscopy observations

Young flower buds (< 4 mm in diameter) were picked and immediately fixed for 24 h in FAA (5% acetic acid, 5% formalin, and 50% ethanol) at 4°C. Following fixation, stamens were dehydrated at 30-min intervals through a 50% step-graded series of ethanol-water solutions, ending with 100% ethanol. Then, the stamens were processed at 30-min intervals through a 50% step-graded series of ethanol-tertiary butyl alcohol (TBA) solutions, ending with 100% TBA. Finally, the stamens were infiltrated over a 24-h period with saturated paraffin-TBA mixtures, and embedded over a 48-h period in paraffin. Lastly, the paraffin blocks were completely polymerized at 4°C.

Then, 10-μm thick sections were cut with a microtome blade R-35 (Feather Safety Razor Co., Ltd Medical Division, Japan) and examined under a stereomicroscope (SZX12, Olympus, Japan). Three blocks were sectioned for each time point, and at least 60 sections were collected for each block. Sections were stained with eosin sodium and observed with a Nikon Eclipse E600 microscope (Nikon Instruments, Japan).

### RNA extraction and preparation

Total RNA was extracted using the Plant RNA Mini Kit (TIANGEN, Inc., China), according to the manufacturer’s instructions. The quality and purity of RNA samples were monitored on 1% agarose gels, and the RNA integrity value (RIN) of samples was assessed using the Agilent 2100 Bioanalyzer (Agilent Technologies, CA, USA) (S1 Fig).

### Construction of cDNA libraries for transcriptome sequencing

A total of 3 μg RNA per sample was used as input material for cDNA library preparation. Sequencing libraries were generated using the NEBNext Ultra^™^ RNA Library Prep Kit for Illumina (NEB, USA), following the manufacturer’s recommendations and index codes were added to attribute sequences to each sample. Briefly, mRNA was purified from total RNA using oligo (dT) beads, and fragmented using divalent cations under elevated temperature in Reaction Buffer (5X). First strand cDNA was synthesized using random hexamer primers. Second strand cDNA synthesis was then performed using DNA Polymerase I and RNase H. The remaining overhangs were converted into blunt ends via a MicroPoly (A) Purist Kit (Ambion, USA). To select cDNA fragments of preferentially 150~200 bp in length, the library fragments were purified with an AMPure XP system (Beckman Coulter, Beverly, USA). Size-selected, adaptor-ligated cDNA was digested with 3 μl USER Enzyme (NEB, USA) at 37°C for 15 min and then at 95°C for 5 min. The fragments were then amplified by PCR using Phusion High-Fidelity DNA Polymerase, Universal PCR Primers, and Index (X) Primer. The library products were purified (AMPure XP system) and library quality was assessed using the Agilent Bioanalyzer 2100 system. Finally, the prepared library was sequenced on an Illumina Hiseq 2000 platform using a paired-end read protocol with 100 bp of data collected per run. RNA-seq data were deposited in the NCBI Sequence Read Archive (SRA) under accession number SRR2192464 and SRR2192489.

### Sequencing data analysis and identification of differentially expressed genes (DEGs)

The raw data obtained were filtered through the standard Illumina pipeline. The filtered reads were further subjected to more stringent quality control using the NGS QC Toolkit (v2.3.2) to remove pair-end reads containing Ns or those that had a PHRED-like quality score of < 20 for at least 10% of the bases [38]. To improve the quality of read mapping, the first nine base pairs of filtered reads, which showed significant deviation of base composition based on the percentages of four different kinds of nucleotides, were trimmed. Then the paired high-quality reads were aligned with the reference genome of *B*. *napus* (http://www.genoscope.cns.fr/brassicanapus/data/) using TopHat v2.0.10 [39].

To identify genes that were differentially expressed between the WSLA and WSLB lines, the gene expression levels were quantified in terms of FPKM (fragments per kilobase of exon per million mapped fragments) using Cufflinks with default parameters [40]. Statistical significance in gene expression between the two samples was assessed using the Cuffdiff program within Cufflinks. The DEGs were identified based on the following thresholds: absolute of log_2_ (fold-change) >1 and *q*-value (false discovery rate (FDR)) <0.01.

### DEGs ontology and bioinformatics analysis

To annotate entire gene sets of the *B*. *napus* genome accurately, a total of 101,040 protein sequences were functionally classified by conducting a homology search against the entire *Arabidopsis thaliana* proteome obtained from the Arabidopsis Information Resource (TAIR) using BLASTP with an e-value cut-off of 1e^-10^ (Unpublished data). Then, we used agriGO (http://bioinfo.cau.edu.cn/agriGO/) and ReviGO (http://revigo.irb.hr/) [41, 42] to identify the putative biological functions and biochemical pathways for DEGs and find statistically overrepresented GO terms. To expand our functional analysis of DEGs involved in RGMS, we visualized biochemical pathway overlays using MapMan software (http://mapman.gabipd.org), as previously described [43].

### Quantitative real-time PCR validation of transcriptome data

To validate the transcriptome data and characterize genes that are differentially expressed between the sterile and fertile oilseed lines, the expression of 93 genes was evaluated by quantitative real-time PCR (RT-qPCR) analysis in the buds of WSLA and WSLB plants at different developmental stages, including large buds (> 4 mm), small buds (< 4 mm), and other components (the stigmas, ovaries, stamens, and anthers) stripped from no pollination floral buds. cDNAs were synthesized according to the manufacturer’s protocol (Takara, Dalian, China) and used as template for RT-qPCR analysis using primers based on the reference *B*. *napus* gene sequences using Primer Premier 5.0 (S1 Table). Real-time PCR was conducted using SYBR Premix *Ex Taq* II (Perfect Real Time) (TaKaRa, China) in a typical 20 μl PCR mixture that included 10 μl of SYBR Premix Ex *Taq* II, 2 μl (100 ng) of template cDNA, and 0.4 μM of each PCR primer. Cycling conditions were 95°C for 2 min, followed by 40 cycles of 95°C for 10 s (denaturation), followed by 60°C for 20 s (annealing and extension). The melting curve of each PCR amplicon was obtained under the following conditions: 95°C for 10 s followed by a constant increase in temperature from 65 to 95°C at an increment of 0.5°C / cycle. Samples were run on the Bio-rad CFX96 Real Time System (USA). Relative expression of the target genes was analyzed with the 2^-ΔΔCt^ method using *BnACTIN7* (At5g09810, EV116054) and *BnUBC21* (At5g25760, EV086936) as internal controls [44]. All samples were amplified in triplicate from the same total RNA preparation and the mean value was used for further analysis. All qRT-PCR assays were repeated three times.

## Results

### Phenotypic characterization of WSLA and WSLB

A phenotypic characterization comparison of the WSLA and WSLB lines showed that the flowers and floral development appeared normal in WSLA (Fig 1A, 1C and 1E), but that the anthers and filaments of WSLA were shorter than those of the WSLB line (Fig 1B and 1F) and the stigmas and ovaries of WSLA were longer than those of WSLB (Fig 1D and 1F). The WSLB plants released mature pollen during the late anther stage, and had a normal floral morphology and architecture, while the WSLA plants failed to produce mature pollen (Fig 1E and 1F). To characterize the cytology of the developing buds in WSLA and WSLB, we performed a histochemical analysis of stamens (< 4 mm) at stage 7 and stage 12, according to the previous report [45]. Eosin sodium staining of transverse sections of stamens revealed that the anthers of the WSLA and WSLB lines differed from each other at both the stage 7 (Fig 1G and 1I) and stage 12 (Fig 1H and 1J). Apparently, only residual defective pollen walls were found in the anthers of the WSLA line (Fig 1G and 1H), indicating that defects in pollen wall structure may underlie the lack of viable pollen in WSLA. Based on these findings, we hypothesized that the defective anthers directly lead to male sterility in WSLA plants. Therefore, we performed a comparative floral transcriptome analysis between WSLA and WSLB to further examine developmental differences at the molecular level.

### Illumina sequencing and mapping of the reference genome

We constructed two cDNA libraries from flower buds of WSLA and WSLB plants and sequenced the cDNA libraries using the Illumina HiSeq 2000 sequencing platform. A total of 57,579,418 and 57,973,385 raw pair-end reads were sequenced in the WSLA (Ste) and WSLB (Fer) libraries, respectively. After the low quality data were filtered out, a total of 111,182,424 clean reads remained in the two libraries; of these, 55,824,023 reads were from WSLA and 55,358,401 were from WSLB. Additionally, > 96% of the reads had an average quality score of >20 (i.e., Q20), and the GC content was consistently 46.18% and 45.66% for WSLA and WSLB, respectively, suggesting that the sequencing was highly accurate. An overview of the Illumina sequencing results and the distribution of distinct clean reads are shown in Table 1.

**Table 1:** Summary of clean Illumina RNA-seq reads for WSLA and WSLB.

Sample	Sequencing NO.	Raw reads	Clean reads	Clean Base (bp)	Error Rate (%)	Q20 (%)	Q30 (%)	GC Content (%)
WSLA	R13100028	57,973,385	55,824,023	8.94G	0.04;0.04	96.40;94.21	90.58;87.80	46.18;46.27
WSLB	R13100027	57,579,418	55,358,401	8.81G	0.04;0.04	96.54;94.38	91.01;88.24	45.66;45.74

Using TopHat software [39], we mapped the reads against the reference genome of *B*. *napus* (http://www.genoscope.cns.fr/brassicanapus/data/) to identify differences in gene expression patterns between the two samples. As shown in Table 2, 86.70%of the total number of clean pair-end reads mapped to multiple (11.10%) or unique (75.60%) genome locations for WSLA, while 86.50% mapped to multiple (11.20%) or unique (75.30%) genome locations for WSLB. Moreover, the total mapped reads could be aligned to each region in the reference genome. Most of the reads mapped to exon regions in both the WSLA and WSLB lines (S2 Fig).

**Table 2:** Summary of Illumina transcriptome sequencing results for WSLA and WSLB.

Reads category	WSLA	WSLB
Total clean reads	55,824,023	55,358,401
Total mapped reads	48399428(86.70%)	47,885,017(86.50%)
Multiple mapped reads	6196466(11.10%)	6,200,141(11.20%)
Uniquely mapped reads	42202961(75.60%)	41,684,876(75.30%)

Numbers in parentheses represent percentages of the total number of clean reads.

### Genes that are differentially expressed between the WSLA and WSLB lines

Using the aforementioned standards, we identified a total of 101,040 genes that were differentially expressed in WSLA and WSLB anthers during development. Moreover, our estimate of the transcript abundance of each gene, which was calculated by analyzing the box-plot distribution of the log FPKM values, suggested that the median and quartile values among the samples being compared for differential expression are almost identical (S3 Fig). To identify differentially expressed genes (DEGs) involved in floral development and flowering with high confidence, we compared the gene expression profiles of the two samples. A rigorous algorithm was developed with an FDR cut-off of ≤ 0.01 and an absolute value of |log_2_Ratio| ≥ 1, which were used as thresholds to judge the significance of differences in transcript abundance [46]. Using these thresholds, we identified 3,199 genes that were differentially expressed between the WSLA and WSLB lines, including 940 genes that were up-regulated and 2,259 genes that were down-regulated in WSLA (S2 and S3 Tables).

### GO enrichment for DEGs

**Fig 2:**
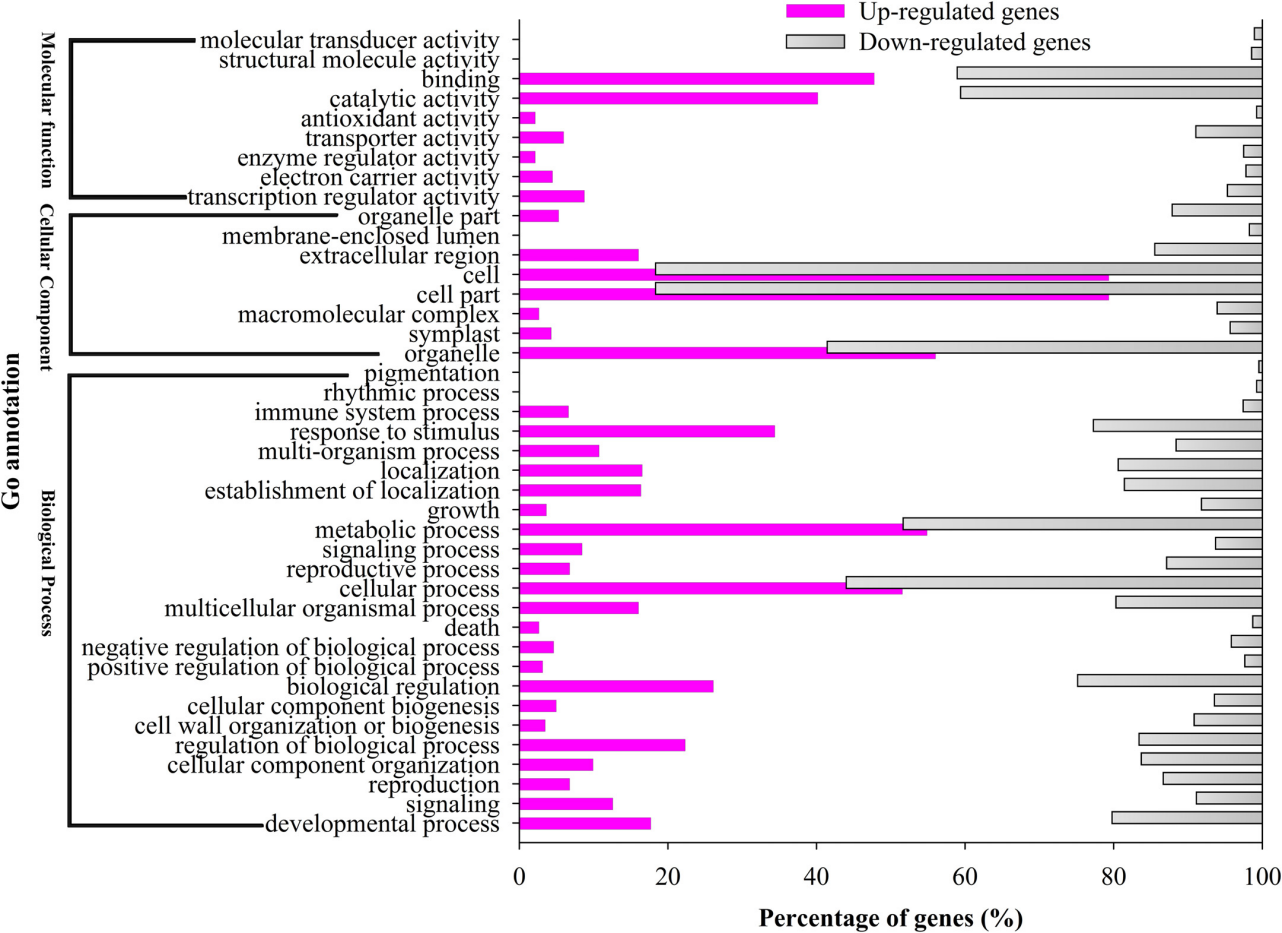
Histogram showing Classification of GO analysis of DEGs in WSLA and WSLB. The Y-axis indicates the sub-categories; the X-axis indicates the percentage of a sub-category of genes in that category. The add purple and gray indicates up- and down-regulated genes in a sub-category.

**Fig 3:**
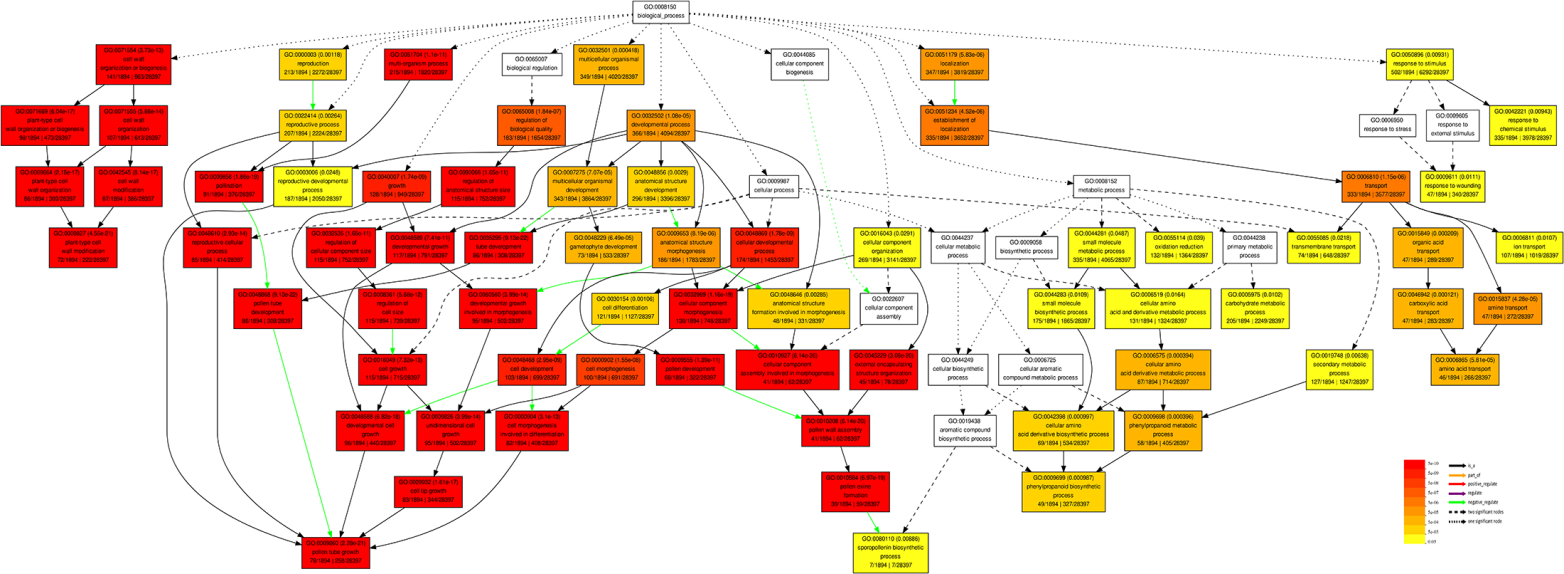
Hierarchical tree graph of overrepresented GO terms in the biological process sub-category. This was found to be enriched in WSLA and WSLB using agriGO (http://bioinfo.cau.edu.cn/agriGO/, Du et al., 2010). Boxes in the graph represent GO terms labeled according to their GO ID, term definition, and statistical information. Significant terms (adjusted *P* ≤ 0.05) are in color (red, orange, or yellow), while non-significant terms are shown as white boxes. In the diagram, the degree of color saturation of a box is positively correlated with the enrichment level of the term. Solid, dashed, and dotted lines represent two, one, and zero enriched terms at both ends connected by the line, respectively. The rank direction of the graph is set from top to bottom.

In this study, a total of 3,199 DEGs were assigned to 41 groups in the following three main GO ontologies: “cellular component”, “molecular function”, and “biological process” (Fig 2). To further evaluate the potential functions of DEGs enriched in male sterility, we assigned gene ontology (GO) categories of biological processes by Singular Enrichment Analysis (SEA) in agriGO (http://bioinfo.cau.edu.cn/agriGO/) [41]. We found that 78 GO terms were in biological process categories, with all terms in these categories being involved in cell wall organization or biogenesis, multi-organism process, modification and assembly, and exine formation. Furthermore, 49 and 12 GO terms were in the cellular component and molecular function categories, respectively (S4 Table). Many of these terms were associated with pollen tube growth, plant-type cell wall modification, pollination, cell tip growth, and reproductive cellular process (Fig 3 and S4 Table). These processes may be directly related to male sterility in WSLA.

**Fig 4:**
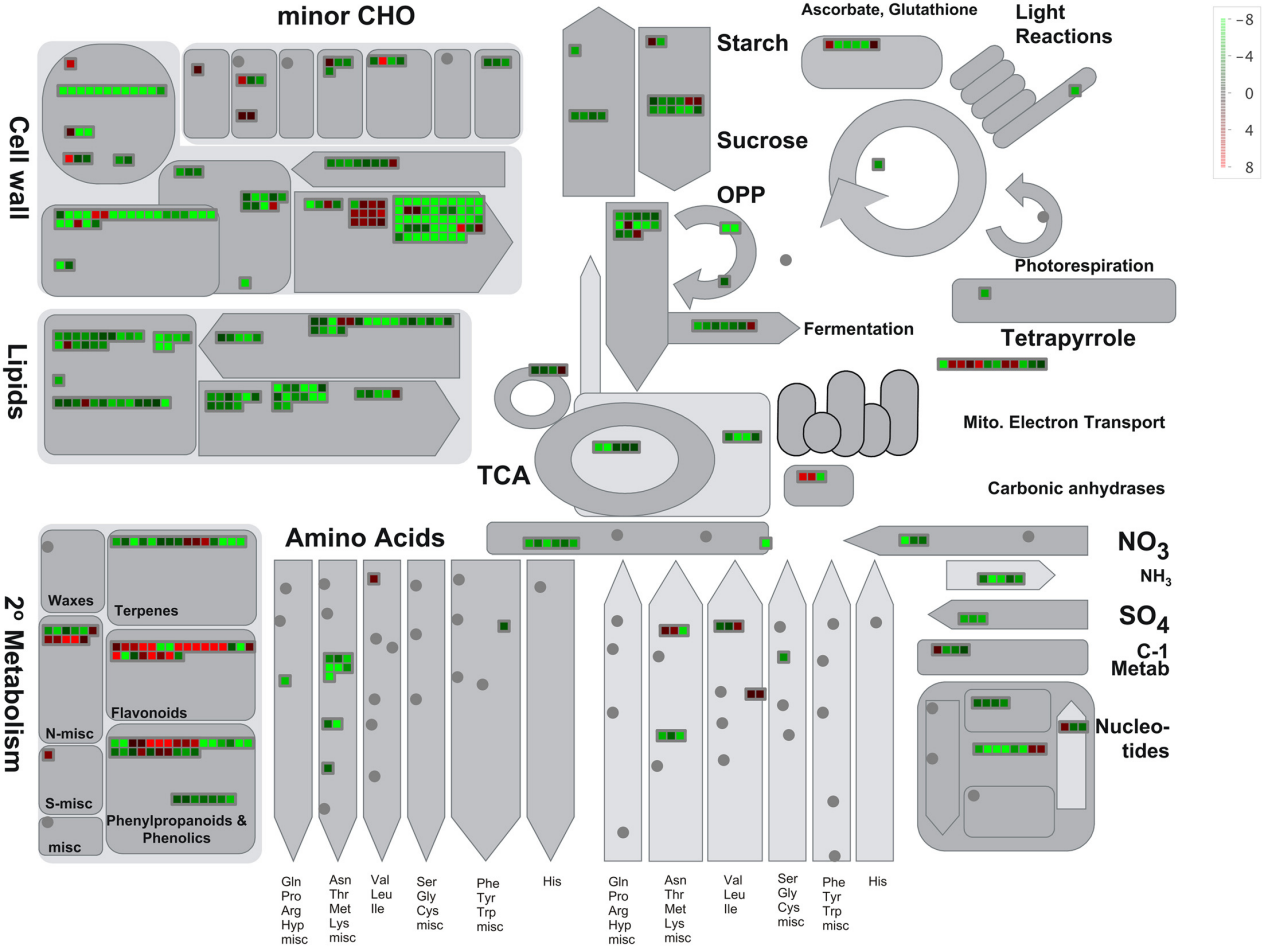
Overview of differentially expressed transcripts involved in different metabolic processes in flowers buds of WSLA. The images were obtained using MapMan and show different functional categories that pass the cutoff (*q*-value < 0.05 and greater than 4-fold change) for differential expression. Each box depicts an individual gene. Up- and down-regulated genes are shown in red and green, respectively. The scale bar represents fold change values.

**Fig 5:**
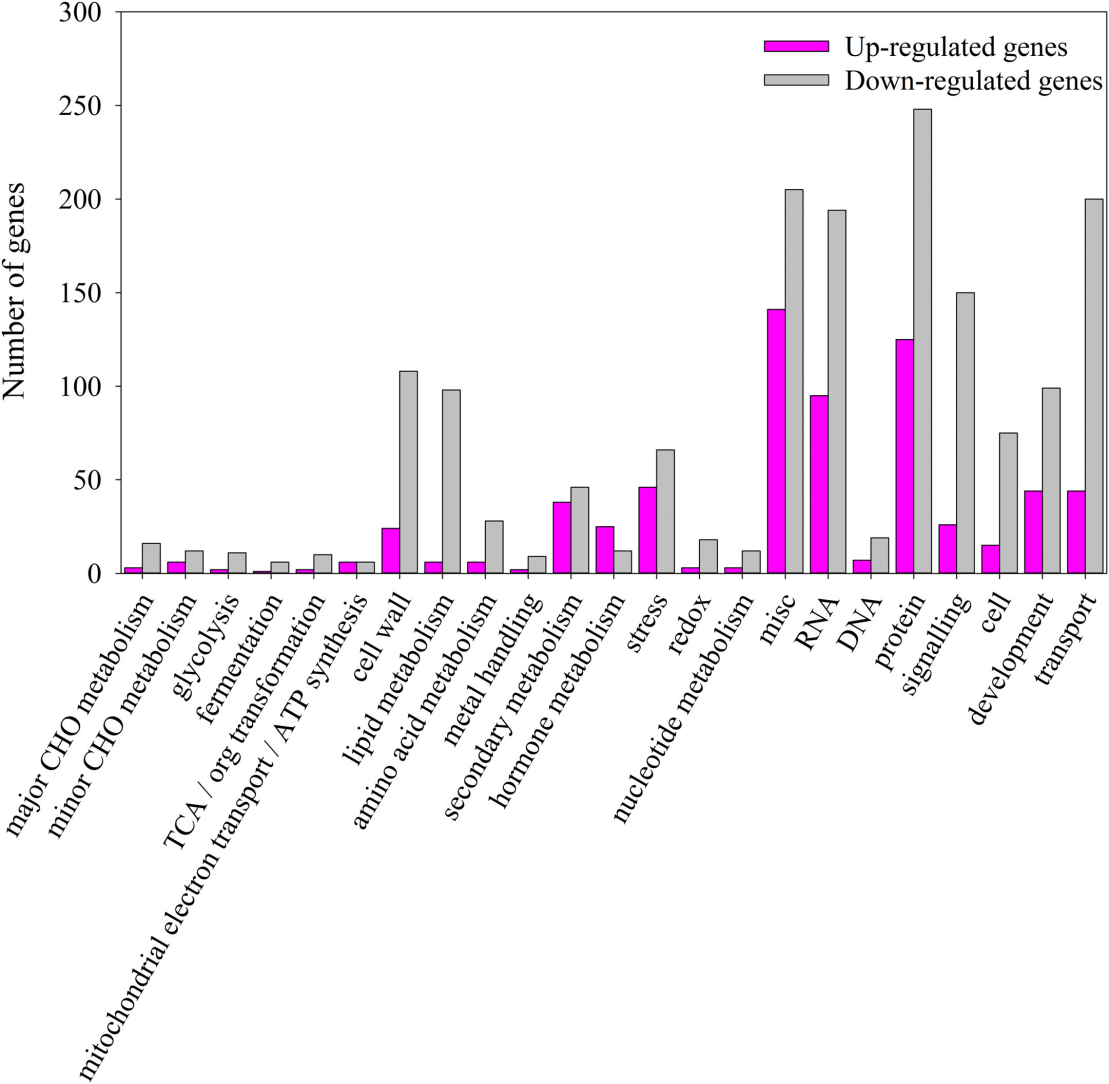
Overview of the total number of DEGs distribution in the changed pathways using the MapMan. The number of Up-regulated and Down-regulated genes within a category is represented by pink and gray bars, respectively. See S7 and S8 Tables for the complete result of the enrichment analysis.

Additionally, we compared the ontologies of genes that were up- and down-regulated in WSLA using semantic similarity-based scatterplots generated in REViGO (http://revigo.irb.hr/). We identified 139 and 231 significantly enriched GO terms (S5 and S6 Tables, Corrected *P*-value < 0.01) associated with DEGs that were up- and down-regulated in WSLA, respectively. GO analysis revealed that most DEGs that were up-regulated in WSLA were involved in pollen exine formation and pollen wall assembly (S4 Fig), whereas those that were down-regulated were mainly involved in cell wall modification and organization and in pollen tube growth (S5 Fig). These results are consistent with the agriGO analysis. To provide an additional level of analysis, we then used the MapMan software tool to view statistically significant male sterile-mediated genes in the context of known metabolic pathways (Fig 4). A prominently infuriation of genes involved in cell wall degradation, ATP synthesis and secondary metabolism, such as Beta-xylosidase genes [47, 48], *MALE STERILITY2* (*MS2*) [28], CYP703A and CYP704Bs [30, 49]. Conversely, genes *ß*-*Ketoacyl-CoA synthases* (KCSs; *KCS7*, and *15*), *Extra Cellular Lipases* (EXLs; *EXL4*, and *6*) and *GLYCINE-RICH PROTEIN* (*GRP18* and *19*), etc., are related to cell wall synthesis, lipid synthesis and degradation were repressed [34, 35]. A complete list of MapMan pathways differentially represented in different metabolic processes (Fig 4) is provided in Fig 5, and S7 and S8 Tables. These results suggest that an intricate mechanism regulates the development of the pollen cell wall in WSLA.

### Reverse transcription quantitative PCR (RT-qPCR) validation of transcriptome results

**Fig 6:**
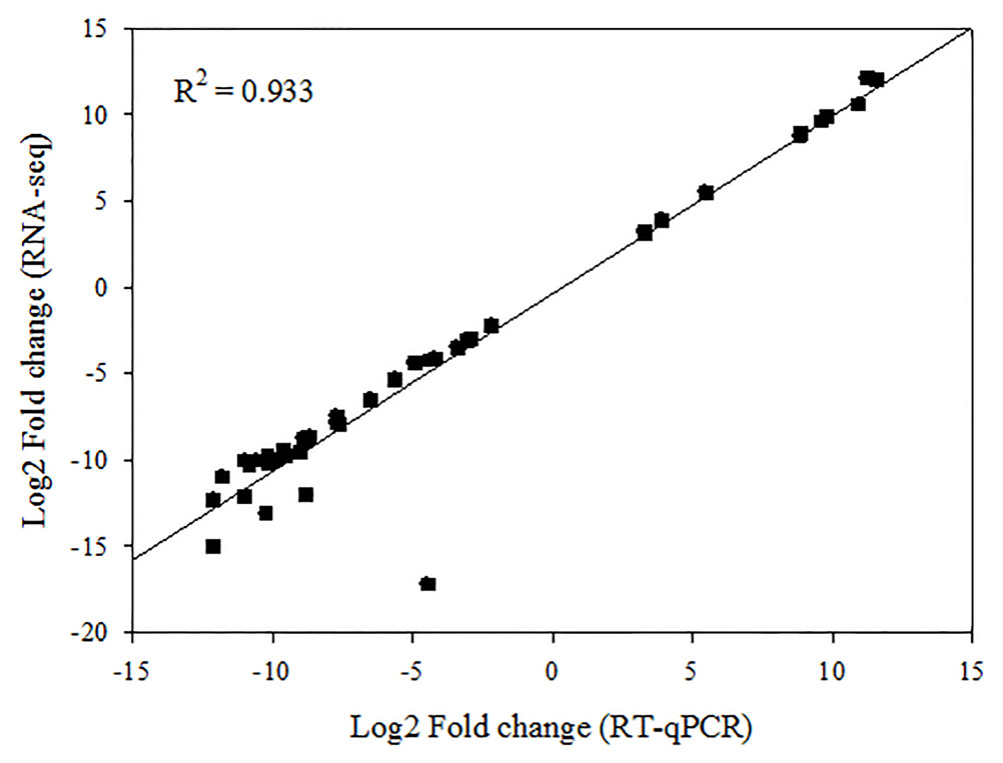
Comparison of gene expression values obtained by RNA-seq and RT-qPCR. Fold changes were calculated for 42 DEGs and a high correlation (R^2^ > 0.93) was observed between the results obtained using the two techniques.

We then used RT-qPCR to verify the DEG data obtained from the transcriptome analysis. Total RNAs were first extracted using the Plant RNA Mini Kit (TIANGEN, China) from the same samples used to construct the cDNA libraries and from other components (the stigmas, ovaries, stamens, and anthers) stripped from no pollination floral buds of WSLA and WSLB. We examined the expression of ninety-three genes with roles in processes such as oxidative phosphorylation, phenylalanine metabolism, starch and sucrose metabolism, fatty acid degradation, and galactose metabolism. Expression is given relative to that in WSLA buds. Then a strong correlation (*R* ^*2*^ > 0.93) was observed between the expression of 42 DEGs detected by RNA-seq and RT-qPCR, which were annotated the function and chromosome positions in *B*. *napus* (Fig 6 and S1 Table). The results indicated that the data were reliable, and we identified the following patterns of expression: 30 genes had high expression in WSLA buds (Group I, S6 Fig); 34 genes had high expression in WSLB buds (Group II, S7 Fig), and 29 genes had decreased expression in WSLA buds and increased expression WSLB buds, with higher expression in the stigmas of WSLA than in WSLB (Group III, S8 Fig). RT-qPCR results were consistent with the expression patterns found in the transcriptome analysis for most of the tested genes, confirming the reproducibility and reliability of the transcriptome data obtained in this study.

### DEGs involved in male sterility in WSLA

We identified several subsets of genes associated with male sterility including the metabolism-related and cell wall-related genes that were previously shown to be involved in anther development.

### Metabolism-related genes

Previous studies showed that basic metabolic pathways, particularly the fatty acid, and phenylpropanoid pathways, are essential for anther development [50, 51].

#### Fatty acids

several genes, identified by RNA-seq analysis, such as *MALE STERILITY2* (*MS1*) [15], *MALE STERILITY2* (*MS2*) [28], *ACYL-COA SYNTHETASE5* (*ACOS5*) [27], *Lipid Transfer Protein 12* (*LTP12*), ß-*Ketoacyl-CoA synthases* (KCSs; *KCS7*, and *15*), and ABC transporters family genes (*WBC27*), etc., which are involved in the fatty acid biosynthesis and metabolism and related to the synthesis of sporopollenin precursors or pollen wall development by regulating lipid acyl metabolism [34, 35]. Genes *LTP12*, *KCS7* and *KCS15* were down-regulated in WSLB, but *MS1*, *MS2*, *ACOS5*, and *WBC27* were up-regulated. Results showed that the abnormal lipid acyl metabolism may be involved in the male sterile in WSLA.

#### Metabolism events

The evidences had found that the anther development was influenced by the basic metabolism events, and numerous genes were identified, such as the CYTOCHROME P450 encoding genes (*CYP703A2*, *CYP704B1*, *CYP705A24*, *CYP86C2*, *CYP86C3*, *CYP98A8*) [29, 30, 49], *SKU5 Similar 13* (*SKS13*), and *ARABIDOPSIS TAPETUM 20* (*TAT20*), which all belong to the Mitochondrial Solute Carrier family [34, 35]; and *POLYKETIDE SYNTHASE A/LAP6* and *B/LAP5* (*PKSA/B*) [33], *TETRAKETIDE a-PYRONE REDUCTASE1* and *2* (*TKPR1/2*) (also called *DIHYDROFLAVONOL 4-REDUCTASE LIKE1* [*DRL1*]) [34], and *Anther-specific protein 6* (*A6*), which are involved in secondary metabolism [34], respectively. Moreover, 19 of the identified DEGs (S2 and S3 Tables) were previously found to be directly regulated by *ABORTED MICROSPORES* (*AMS*) [34]. Six of these DEGs, including *A6*, *WBC27*, *LAP5*, *DRL1*, *CYP703A2*, and *CYP704B1* (S2 Table), were up-regulated in WSLA and associated with secretion of sporopollenin during flowering. In addition, *MS1*, *MS2*, *LAP6*, and *ACOS5*, which are involved in anther development [15, 19, 22], were also up-regulated in WSLA (S2 Table). Absolutely, all these genes were also associated with the constituents of the pollen wall development. So they are apparently up-regulated in WSLA might result in abundant sporopollenin accumulation and male sterility. Unlike the *EMS1/EXS*, *MS1*, and *AMS*, the WSLA contribute to male sterility via different mechanisms.

### Pollen wall formation

A subsets genes involved in pollen wall formation were identified. Down-regulated in WSLA were two *Extra Cellular Lipases* (EXLs; *EXL4*, and *6*) and *GLYCINE-RICH PROTEIN* (*GRP18* and *19*), which were implicated in the pollen exine and pollen wall formation and subsequent pollination [34]. In addition, the evidence showed that the *CYP98A8* and its paralog *CYP98A9* catalyzed oxygenation of phenol-amides, which is crucial for pollen wall [52]. Moreover, the SEM results showed that the WSLA displayed the defective pollen walls (Fig 1G and 1H), indicating that the abnormal pollen wall was associated with the down-regulated gene. But not all of the genes involved in pollen wall formation showed significantly different between the WSLA and WSLB, such as genes *AMS* [34, 35], *CYP98A9* [52], therefore, the mechanism of male sterile in WSLA need further to study.

### Stigma-specific genes

The stigma providing the nutrients and signals for pollen grains, which is crucial for the process of pollination [53]. Interestingly, the stigma-specific genes, such as *AT4G23690*, *AT2G34810*, *AT2G39350*, *AT1G22990*, *AT1G22990*, and *AT3G13790* (S2 Table), were also dramatically up-regulated in WSLA using the RNA-seq, which could account for the larger stigmas observed in this line (Fig 1D and 1F). Functional specialization of these genes will provide new insight into the mechanism of pollen–stigma interactions.

### The triplication of genes involved in male sterility

The *Brassicas* plants have the same ancestry with a propensity of genome duplications and mergers in evolutionary process [54]. The allotetraploid species *B*. *napus* was derived from interspecific hybridization of two diploid species, *B*. *rapa* and *B*. *oleracea*, and the excessive gene loss is typical after polyploidy formation in eukaryotes [55]. In this study, all copies of DEGs verified by RT-qPCR were identified, and each of the orthologous blocks corresponding to ancestral blocks was identified using collinearity between orthologues on the genome of *B*. *rapa*, *B*. *oleracea*, *B*. *napus*, and *A*. *thaliana* genome (S9 Table). These results provides a chance to study gene retention in triplicated genomes. Overall, most of the identified DEGs play important roles in flowering, and would therefore enable a better understanding of gene expression patterns during flowering in *B*. *napus*. Importantly, these DEGs provide clues as to the identities of genes involved in RGMS.

## Discussion

Male sterile plants are useful tools for hybrid seed production. Pollen grains are a prerequisite for propagation in flowering plants. To identify the genes that are differentially expressed during RGMS, we performed transcriptome profiling of WSLA and WSLB buds. A sequencing depth of 5.8 million tags per library was reached (Table 2). Using the *B*. *napus* as reference (http://www.genoscope.cns.fr/brassicanapus/data/) [54], a total of 3,199 DEGs (*P* <0.01) were detected between the WSLA and WSLB lines, with 940 being up-regulated and 2,259 being down-regulated in WSLA (S2 and S3 Tables). Based on a GO functional analysis, we grouped the DEGs into 25 functional categories, including cell wall biogenesis, metabolic process, pollination, and response to stimulus (Fig 2). These findings indicate that male sterility is a complex polygenic trait.

In this study, both the WSLA and WSLB lines displayed normal flowers, but the pollen walls were degraded in WSLA (Fig 1). Hence, further studies should focus on identifying the defective metabolic events involved in pollen wall formation in the mutant. Recently, biochemical and genetic studies showed that *AMS* regulates the biosynthesis of lipidic, phenolic, and flavonoid compounds and is an important regulator of tapetal development and pollen wall formation in *Arabidopsis* [34]. Transcriptome analysis revealed that 48 genes were down-regulated in *ams* buds, and 6 of these genes were previously reported to be involved in anther development [15, 19, 22, 35]. We found that homologues of *AMS* were differentially expressed in WSLA and WSLB lines, and that *ß*-*Ketoacyl-CoA synthases* (*KCSs*, *KCS7* and *15*); *lipid transfer protein* (*LTP*) and *Extra Cellular lipases* (*EXLs*; *EXL4* and *EXL6*), two *GLYCINE-RICH PROTEIN* encoding genes (*GRP18* and *19*) (S3 Table) implicated in lipid acyl metabolism; and 13 genes (such as *CYP86C3*, *CYP705A24*, *CYP98A8*, *CYP86C2*, and *PAP6*) encoding miscellaneous enzymes (S3 Table) were down-regulated in WSLA. These down-regulated genes were validated using RT-qPCR (S7 Fig), and indicate that similar biological pathways may be involved in male sterility in the WSLA line and in the *ams Arabidopsis* mutant.

Sporopollenin is an essential component of exine and is mainly composed of fatty acids and phenols [23], the biosynthesis of which is regulated by many genes (Aarts et al., 1997, Ariizumi et al., 2004, Chen et al., 2011, Dobritsa et al., 2009b, Li et al., 2010, Shi et al., 2011). Four genes involved in lipid acyl metabolism (*MS2*, *ACOS5*, *WBC27* and *At5g07230* [*LTP*]), four encoding miscellaneous enzymes (*CYP703A2*, *CYP704B1*, *ATA1*, and *A6*), and four involved in secondary metabolism (*LAP5*, *LAP6*, *CCRL6*, and *DRL1*) were up-regulated in WSLA (S2 Table and S6 Fig), whereas homologous genes were significantly down-regulated in *ams* buds [34, 35]. *MS2*, a fatty acyl reductase associated with sporopollenin biosynthesis, and *ACOS5*, a medium-chain fatty acyl-COA involved in pollen wall development, were also down-regulated in *ams* mutants [24, 27]. We also found that the ABC transporter *WBC27*, which is associated with pollen wall development [34, 35], was dramatically up-regulated in WSLA (S6 Fig). Multi-functional enzymes (LAP5 and LAP6) catalyze the biosynthesis of phenolic constituents of sporopollenin, and *LAP5* is involved in fatty acid hydroxylation (*CYP703A2*) [33], exerting a synergistic effect with *WBC27* and *MS2* [35]. Furthermore, previous results showed that *MS1* is critical for tapetal and pollen wall formation [15]. All of these genes were expressed at higher levels in WSLA than in WSLB (S2 Table and S6 Fig), suggesting that the excessive accumulation of sporopollenin and abnormal fatty acids might result in RGMS. This research, although it has not identified the candidate gene directly involved in the process of male sterility, provides insight into the molecular mechanisms underlying RGMS in WSLA in *B*. *napus*.

The stigma plays a critical role in the sexual reproduction of flowering plants. It inhibits the adhesion, hydration, and germination of foreign pollen [53, 56]. Identifying stigma-specific genes is useful for better understanding pollen–stigma communication and pollen tube guidance. Interestingly, we identified a group of genes that exhibited higher expression in the stigmas of WSLA plants (S2 Table and S8 Fig), consistent with findings presented in Swanson et al. (2005). Increased expression of these genes may result in larger stigmas in WSLA plans, but the underlying molecular mechanism remains to be elucidated.

Male sterility is a complex biological process that is regulated by numerous genes. In this study, we identified a large number of genes that are differentially expressed in the buds of developing WSLA and WSLB plants, and verified the expression of these genes by RT-qPCR. Based on these results, these genes involved in the secretion of sporopollenin showed higher expression level in WSLA than WSLB, which leads to the excess sporopollenin accumulation in WSLA plants. Therefore, we propose that the abundant sporopollenin with the defects in fatty acid biosynthesis during the early stages of bud development that result in the formation of defective pollen walls, which leads to the male sterility of WSLA plants.

### Conclusions

Using a high-throughput sequencing approach, we conducted a comprehensive analysis of fertile and sterile *B*. *napus* lines, the latter of which exhibits RGMS. We identified numerous DEGs as potential candidates involved in RGMS. Further functional analysis of these genes will help us to well understand the mechanism underlying RGMS in *B*. *napus*. Additionally, this resource will provide a solid foundation for developing molecular tools that can be used in breeding programs to enhance rapeseed yield.

## Supporting Information

S1 Fig RNA quality determined with the Agilent 2100 Bioanalyzer. (DOCX)

S2 Fig The percentage of Illumina sequencing reads mapped to reference genome regions in the WSLA and WSLB libraries. (DOCX)

S3 Fig Boxplot of the log FPKM (fragments per kilobase of exon per million fragments mapped) expression values in WSLA and WSLB. The figure shows the boxplot of the log FPKM values in the two libraries. The plot shows that the median of the FPKM values across the libraries being compared for differential expression are comparable.
(DOCX)

S4 Fig Network of GO enrichment of the up-regulated DEGs in WSLA, using REViGO (http://revigo.irb.hr/). Bubble color indicates the user-provided *p*-value (red present –log(p-value)> 10, white present –log(p-value >2). Other means of thresholds were defined by the published reference described (Supek et al. 2011).
(DOCX)

S5 Fig Network of GO enrichment of the down-regulated DEGs in WSLA, using REViGO (http://revigo.irb.hr/). Bubble color indicates the user-provided *p*-value (red present –log(p-value)> 10, white present –log(p-value >2). Other means of thresholds were defined by the published reference described (Supek et al. 2011).
(DOCX)

S6 Fig RT-qPCR verification of DEGs (Group I). S/F denotes sterile sample (WSLA) and F denotes fertile sample (WSLB); Bu1, large buds (> 4 mm); Bu2, small buds (< 4 mm); St: stigmas; Ov: ovaries; S-A: stamens and anthers. Values represent the average ± SD of three biological replicates with three technical replicates of per samples.
(DOCX)

S7 Fig RT-qPCR verification of DEGs (Group II). S/F denotes sterile sample (WSLA) and F denotes fertile sample (WSLB); Bu1: large buds (> 4 mm); Bu2: small buds (< 4 mm); St: stigmas; Ov: ovaries; S-A: stamens and anthers. Values represent the average ± SD of three biological replicates with three technical replicates of per samples.
(DOCX)

S8 Fig RT-qPCR verification of DEGs (Group III). S/F denotes sterile sample (WSLA) and F denotes fertile sample (WSLB); Bu1: large buds (> 4 mm); Bu2: small buds (< 4 mm); St: stigmas; Ov: ovaries; S-A: stamens and anthers. Values represent the average ± SD of three biological replicates with three technical replicates of per samples.
(DOCX)

S1 Table Primer sequences of 93 DEGs used to for RT-qPCR analysis between the WSLA and WSLB lines. (XLSX)

S2 Table List of up-regulated DEGs identified in WSLA. (XLSX)

S3 Table List of down-regulated DEGs identified in WSLA. (XLSX)

S4 Table Gene ontology (GO) classes potentially involved in WSLA. (XLSX)

S5 Table List of significantly enriched GO terms in up-regulated DEGs. (XLSX)

S6 Table List of significantly enriched GO terms in down-regulated DEGs. (XLSX)

S7 Table MapMan Bins of Up-regulated genes enriched in different metabolic processes. (XLSX)

S8 Table MapMan Bins Down-regulated genes enriched in different metabolic processes. (XLSX)

S9 Table The different copies of DEGs in the *B*. *rapa*, *B*. *oleracea* and *B*.*napus* genome. (XLSX)
