# Supplementary figures and images for: Comparative Transcriptome Analysis of Recessive Male Sterility (RGMS) in Sterile and Fertile Brassica napus Lines
Source: PLoS One. 2015 Dec 10;10(12):e0144118. doi: 10.1371/journal.pone.0144118 (PMC4675519; doi:10.1371/journal.pone.0144118)

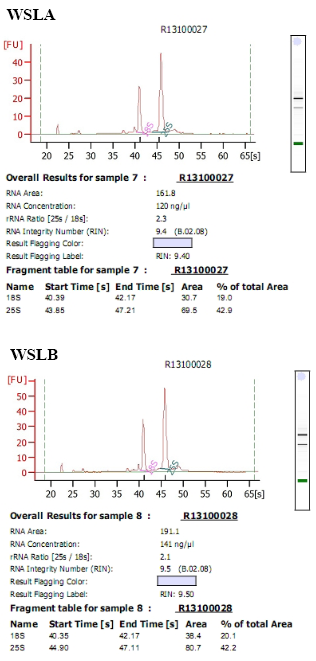


**S1 Fig. RNA quality determined with the Agilent 2100 Bioanalyzer.**

Supplement: S1 Fig — (DOCX) [file pone.0144118.s001.docx]
