# Supplementary material for: Comparative Transcriptome Analysis of Recessive Male Sterility (RGMS) in Sterile and Fertile Brassica napus Lines
Source: PLoS One. 2015 Dec 10;10(12):e0144118. doi: 10.1371/journal.pone.0144118 (PMC4675519; doi:10.1371/journal.pone.0144118)
Supplement: S2 Fig — (DOCX) [file pone.0144118.s002.docx]

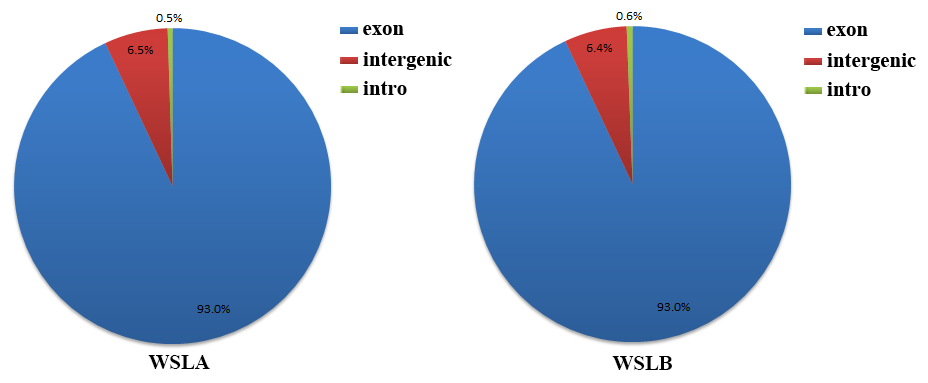


**S2 Fig. The percentage of Illumina sequencing reads mapped to reference genome regions in the WSLA and WSLB libraries.**
