# Supplementary material for: Comparative Transcriptome Analysis of Recessive Male Sterility (RGMS) in Sterile and Fertile Brassica napus Lines
Source: PLoS One. 2015 Dec 10;10(12):e0144118. doi: 10.1371/journal.pone.0144118 (PMC4675519; doi:10.1371/journal.pone.0144118)
Supplement: S3 Fig — The figure shows the boxplot of the log FPKM values in the two libraries. The plot shows that the median of the FPKM values across the libraries being compared for differential expression are comparable. (DOCX) [file pone.0144118.s003.docx]

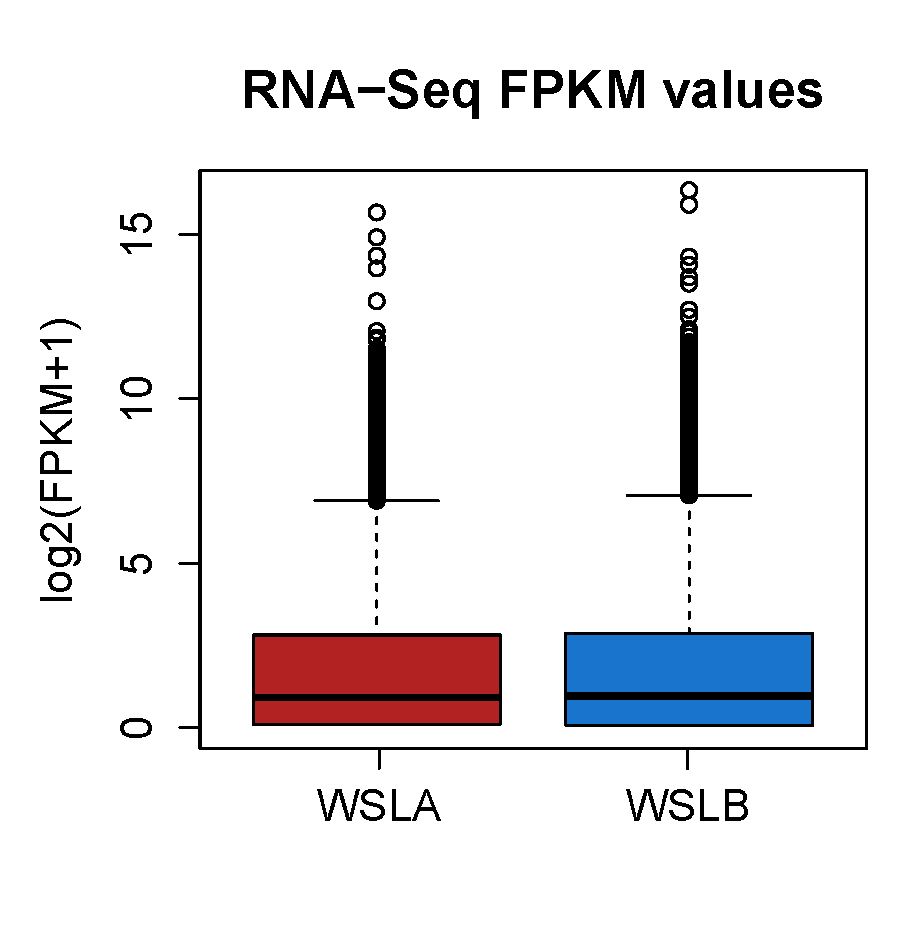


**S3 Fig.** **Boxplot of the log FPKM (fragments per kilobase of exon per million fragments mapped) expression values in WSLA and WSLB.**

The figure shows the boxplot of the log FPKM values in the two libraries. The plot shows that the median of the FPKM values across the libraries being compared for differential expression are comparable.
