# Supplementary material for: Comparative Transcriptome Analysis of Recessive Male Sterility (RGMS) in Sterile and Fertile Brassica napus Lines
Source: PLoS One. 2015 Dec 10;10(12):e0144118. doi: 10.1371/journal.pone.0144118 (PMC4675519; doi:10.1371/journal.pone.0144118)
Supplement: S4 Fig — Bubble color indicates the user-provided p-value (red present –log(p-value)> 10, white present –log(p-value >2). Other means of thresholds were defined by the published reference described (Supek et al. 2011). (DOCX) [file pone.0144118.s004.docx]

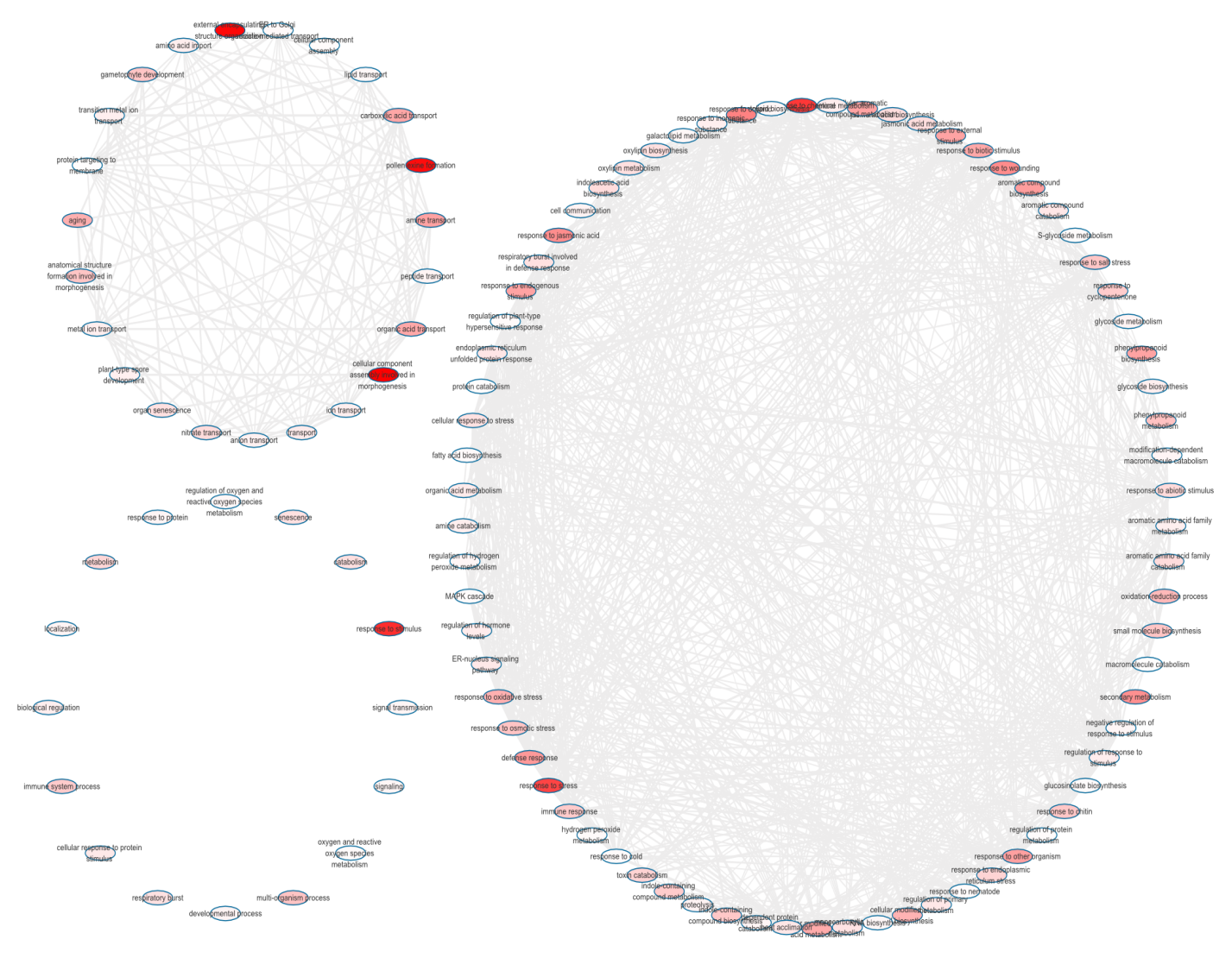


**S4 Fig. Network of GO enrichment of the up-regulated DEGs in WSLA using REViGO (http://revigo.irb.hr/).**

Bubble color indicates the user-provided *p*-value (red present –log(p-value)> 10, white present –log(p-value >2). Other means of thresholds were defined by the published reference described (Supek et al. 2011).
