# Supplementary material for: Comparative Transcriptome Analysis of Recessive Male Sterility (RGMS) in Sterile and Fertile Brassica napus Lines
Source: PLoS One. 2015 Dec 10;10(12):e0144118. doi: 10.1371/journal.pone.0144118 (PMC4675519; doi:10.1371/journal.pone.0144118)
Supplement: S7 Fig — S/F denotes sterile sample (WSLA) and F denotes fertile sample (WSLB); Bu1: large buds (> 4 mm); Bu2: small buds (< 4 mm); St: stigmas; Ov: ovaries; S-A: stamens and anthers. Values represent the average ± SD of three biological replicates with three technical replicates of per samples. (DOCX) [file pone.0144118.s007.docx]

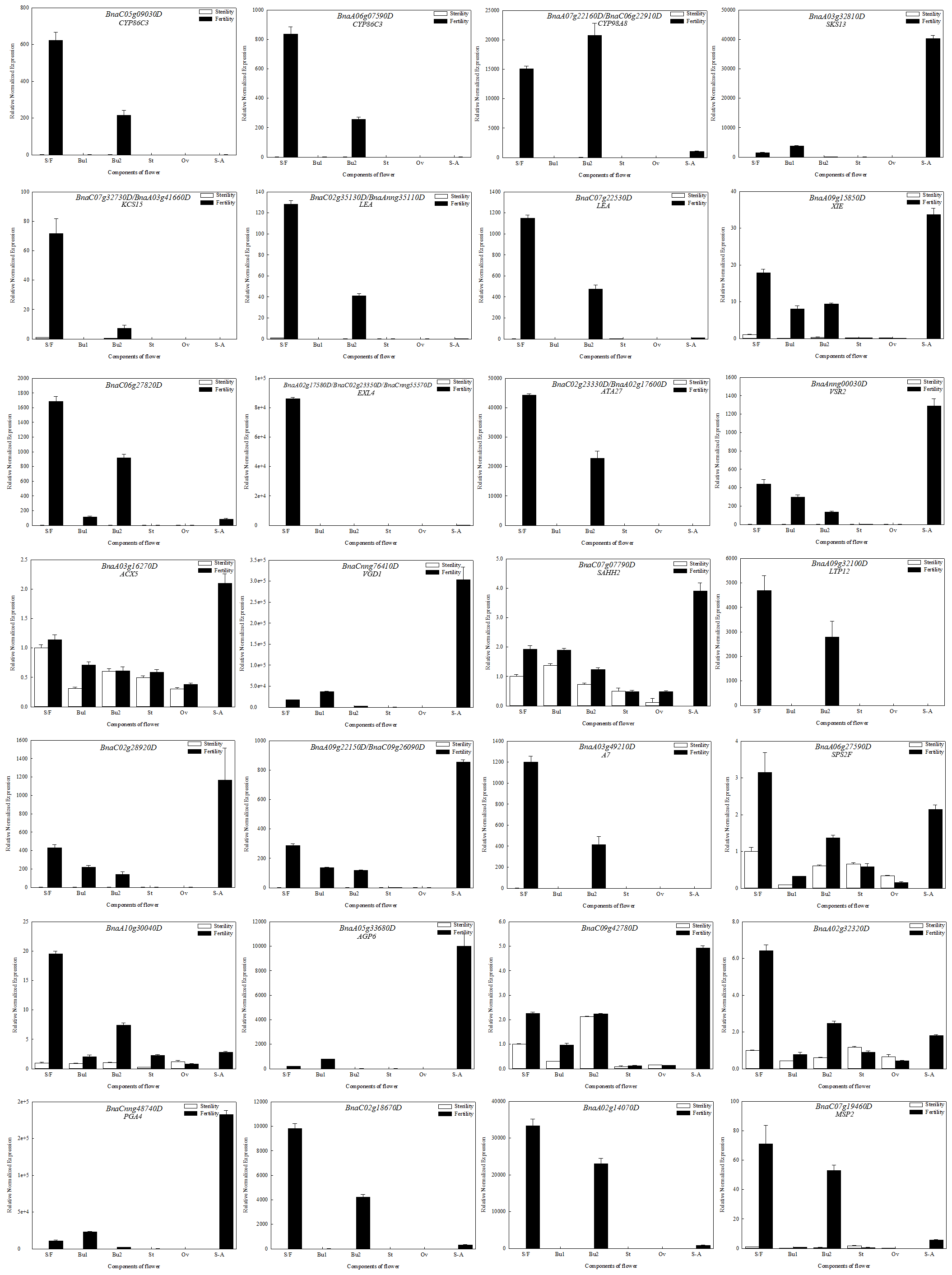


**S7 Fig. RT-qPCR** **verification of DEGs (Group II).**

S/F denotes sterile sample (WSLA) and F denotes fertile sample (WSLB); Bu1: large buds (> 4 mm); Bu2: small buds (< 4 mm); St: stigmas; Ov: ovaries; S-A: stamens and anthers. Values represent the average ± SD of three biological replicates with three technical replicates of per samples.
